# Retraction: The Downregulation of GFI1 by the EZH2-NDY1/KDM2B-JARID2 Axis and by Human Cytomegalovirus (HCMV) Associated Factors Allows the Activation of the HCMV Major IE Promoter and the Transition to Productive Infection

**DOI:** 10.1371/journal.ppat.1013827

**Published:** 2026-01-05

**Authors:** The PLOS Pathogens Editors

Following the publication of this article [1], concerns were raised about Figs 1–3 and S2.

Specifically,

- In Fig 1, the actin panel for PHF2 in Fig 1A appears similar to the actin panel for JMJD3 in Fig 1D
- In Fig 2C, bands in lanes 1–4 of the upper actin panel appear similar to bands in lanes 1–4 of the lower actin panel, and bands in lanes 1–3 of the upper and lower actin panels appear similar to bands in lanes 6–8 of the lower actin panel
- In Fig 3C, panels mtMIEP/shEZH2 and mtMIEP/shJARID2 appear to partially overlap
- In Fig 3C, panels mtMIEP/GFI1 and wtMIEP/shGFI1 appear to partially overlap
- In Fig S2A, pLKO.1 and pBabe panels appear similar
- The JMJD3 panel in Fig S2B appears to show the same data as the shNDY1/KDM2B DAPI and EI1 panels in Fig S2A

The authors provided underlying images and replacement figures in response to these concerns, but the data provided did not fully resolve the concerns about the published results.

In light of the above concerns that question the reliability and integrity of the results presented in [1], the *PLOS Pathogens* Editors retract this article.

GS and PNT did not agree with the retraction. IS responded but expressed neither agreement nor disagreement with the editorial decision. AM, IC, SAE, CD, SCK, and FK either did not respond directly or could not be reached.
